# Microangiopathic hemolytic anemia associated with metastatic breast cancer: case report and literature review

**DOI:** 10.1186/s40064-016-2312-4

**Published:** 2016-05-20

**Authors:** Daisuke Takabatake, Kazuyuki Oishi

**Affiliations:** Breast and Thyroid Surgery, Kochi Health Science Center, 2125-1 Ike, Kochi, 781-8555 Japan

**Keywords:** Microangiopathic hemolytic anemia (MAHA), Breast cancer, Thorombotic microangiopathy (TMA)

## Abstract

**Introduction:**

Microangiopathic hemolytic anemia (MAHA) is a mechanical hemolytic anemia characterized by the emergence of fragmented red cells in peripheral blood. Here, we report a case of breast cancer associated with cancer-related (CR)-MAHA along with a literature review.

**Case description:**

The patient was a 54-year-old woman who made an emergency visit to our hospital because of low back pain, shoulder pain, visual impairment, and anemia. She was diagnosed with stage IV, ER-positive, PgR-positive, HER2-negative left breast cancer (invasive lobular carcinoma), with left axillary adenopathy, metastasis to the soft tissue of the orbital region, multiple bone metastases, pleural dissemination, and metastasis to the stomach and para-aortic lymph nodes. Chemotherapy was initiated successfully; tumor marker levels normalized and the visceral metastases almost disappeared. Hormone therapy was administered for maintenance. Two and a half years later, rapid elevation in tumor marker levels and severe anemia were noted, and fragmented red cells and poikilocytes emerged in the peripheral blood. Positron emission tomography–computed tomography and bone scintigraphy revealed multiple bone metastases, but no evidence of visceral metastasis. CR-MAHA associated with multiple bone metastases was diagnosed, and Paclitaxel chemotherapy was initiated with frequent blood transfusions. Her anemia gradually improved, with a decrease in tumor marker levels and the number of blood transfusions. Three months later, tumor marker levels increased again. Because the anemia was also exacerbated, chemotherapy was changed to eribulin. Tumor marker levels temporally decreased, and the anemia tended to improve, but 3 months later, the levels were elevated again and the anemia was exacerbated. A switch to another regimen was planned, but best supportive care was chosen instead because of rapid deterioration of liver function. The patient died a month later.

**Discussion and evaluation:**

CR-MAHA is thought to have a different pathologic mechanism from TTP or HUS. Although CR-MAHA is a clinical condition associated with a very poor prognosis, we consider it controllable for long period by rapid introduction of chemotherapy in many cases.

**Conclusions:**

CR-MAHA is a nearly oncologic emergency that medical oncologists need to be able to recognize even though it rarely occurs in breast cancer.

## Background

Thorombotic microangiopathy (TMA) describes a specific pathologic lesion in which abnormalities in the vessel wall of arterioles and capillaries lead to microvascular thrombosis. The initial evaluation of a patient with suspected TMA is very important, because many patients require directed therapy for the underlying disorder rather than specific therapy for TMA, and the directed therapies vary depending on the disorder (James and Carla 2014). Microangiopathic hemolytic anemia (MAHA) is one of the major characteristic symptoms of TMA (James and Carla 2014). MAHA is a descriptive term for non-immune hemolytic anemia characterized by the fragmentation of red blood cells due to microangiopathy caused by a variety of underlying diseases (James and Carla 2014). Currently, no clear diagnostic criteria exist, but the emergence of fragmented red cells and schistocytes in peripheral blood marks MAHA. Characteristic laboratory data are a negative direct antiglobulin (Coombs) test, an increased lactate dehydrogenase (LDH) level, increased indirect bilirubin, and low haptoglobin.

MAHA can present with various systemic disorders including thrombotic thrombocytopenic purpura (TTP), hemolytic uremic syndrome (HUS), disseminated intravascular coagulation (DIC), systemic infections, and autoimmune disorders. Its occurrence in cases of widespread metastasis of malignant tumors [cancer-related MAHA (CR-MAHA)] has also been reported, albeit rarely (Pendse et al. 2014; Lechner and Obermeier 2012; Shin et al. 2011; Rauh et al. 2011; George 2011; Himmelmann and Schefer 2009; Ali et al. 2007; Arkenau et al. 2005; Yeh et al. 2002; Lockhart 2001; Fontana et al. 2001; Ataga and Graham 1999; Narita et al. 1997; Tsatsaris et al. 1996; Nordstrom and Strang 1993; Bastecky et al. 1992; Collins et al. 1991; Canellos and Mark 1984). CR-MAHA has been reported in several types of carcinoma (Pendse et al. 2014; Lechner and Obermeier 2012; Shin et al. 2011; Rauh et al. 2011; George 2011; Himmelmann and Schefer 2009; Ali et al. 2007; Arkenau et al. 2005; Yeh et al. 2002; Lockhart 2001; Fontana et al. 2001; Ataga and Graham 1999; Narita et al. 1997; Tsatsaris et al. 1996; Nordstrom and Strang 1993; Bastecky et al. 1992; Collins et al. 1991; Canellos and Mark 1984), but its pathophysiology remains largely unknown and there is no established management method. In general, its prognosis is said to be very poor, but many reports indicate that the patients respond to drug therapy (Lechner and Obermeier 2012; Lockhart 2001; Fontana et al. 2001; Narita et al. 1997; Nordstrom and Strang 1993; Collins et al. 1991). Here, we report a case of metastatic breast cancer associated with MAHA, which rapidly progressed. Furthermore, we conducted a literature review on cases of breast cancer with MAHA and studied the pathology of CR-MAHA and its appropriate management.

## Case description

The patient was a 54-year-old woman who made an emergency visit to our hospital because of low back pain, shoulder pain, visual impairment, and anemia. A soft mass in the right orbital adipose tissue, thickening of the entire circumference of the wall of the gastric corpus, left axillary adenopathy, multiple nodular shadows in the bilateral pleura, pleural effusion, ascites, and a swollen para-aortic lymph nodes were found on computed tomography (CT). Furthermore, bone scintigraphy showed uptake in multiple sites including the cranial bone, spine, and ribs.

At first, systemic metastasis and multiple bone metastases of an occult cancer was considered, and excisional biopsy of the axillary lymph node was performed for a definitive diagnosis. Based on the pathological findings, axillary lymph node metastasis of invasive lobular carcinoma (ER-positive, PgR-positive, and HER2-negative) was diagnosed. A mass was not palpable in the left breast, and no clear mass shadow was observed within the left breast on ultrasonography (US), but there were irregular low echoic lesions and structural distortions in the upper-lateral quadrant of the breast.

Core-needle biopsy yielded a diagnosis of invasive lobular carcinoma (stage IV) originating from primary left breast cancer was diagnosed. Levels of the tumor markers CEA and CA15-3 were elevated to 26.6 ng/ml and 90.8 U/ml, respectively. Chemotherapy (epirubicin 90 mg/m^2^ + cyclophosphamide 600 mg/m^2^) was initiated. The tumor marker levels normalized 6 months later, and the visceral metastases almost disappeared on imaging a year later.

**Fig. 1:**
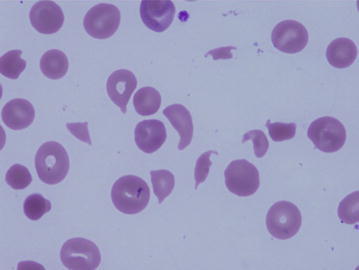
Fragmented red cell had appeared in the peripheral blood and presented severe hemolytic anemia

After performing ten cycles of chemotherapy, the treatment was switched to hormone therapy with letrozole for maintenance. Rapid elevation of tumor marker levels (CEA 3.9–23.0, CA15-3 9.0–38.7), as manifested by severe anemia, was noted 14 months later, and fragmented red cells emerged in the peripheral blood (Fig. 1). The Laboratory data were as follows, Red Blood Cell counts 1.51 × 10^6^/µl, Hemoglobin 5.4 g/dl, Platelet counts 8.0 × 10^4^ µl, LDH 1137 IU/µl. The result of Coombs’ test was negative, no coagulation system abnormalities were noted (D-dimer 1.7 µg/ml, FDP 3.7 μg/ml, PT-INR 1.02, and APTT 31 s), and renal function was normal.

**Fig. 2:**
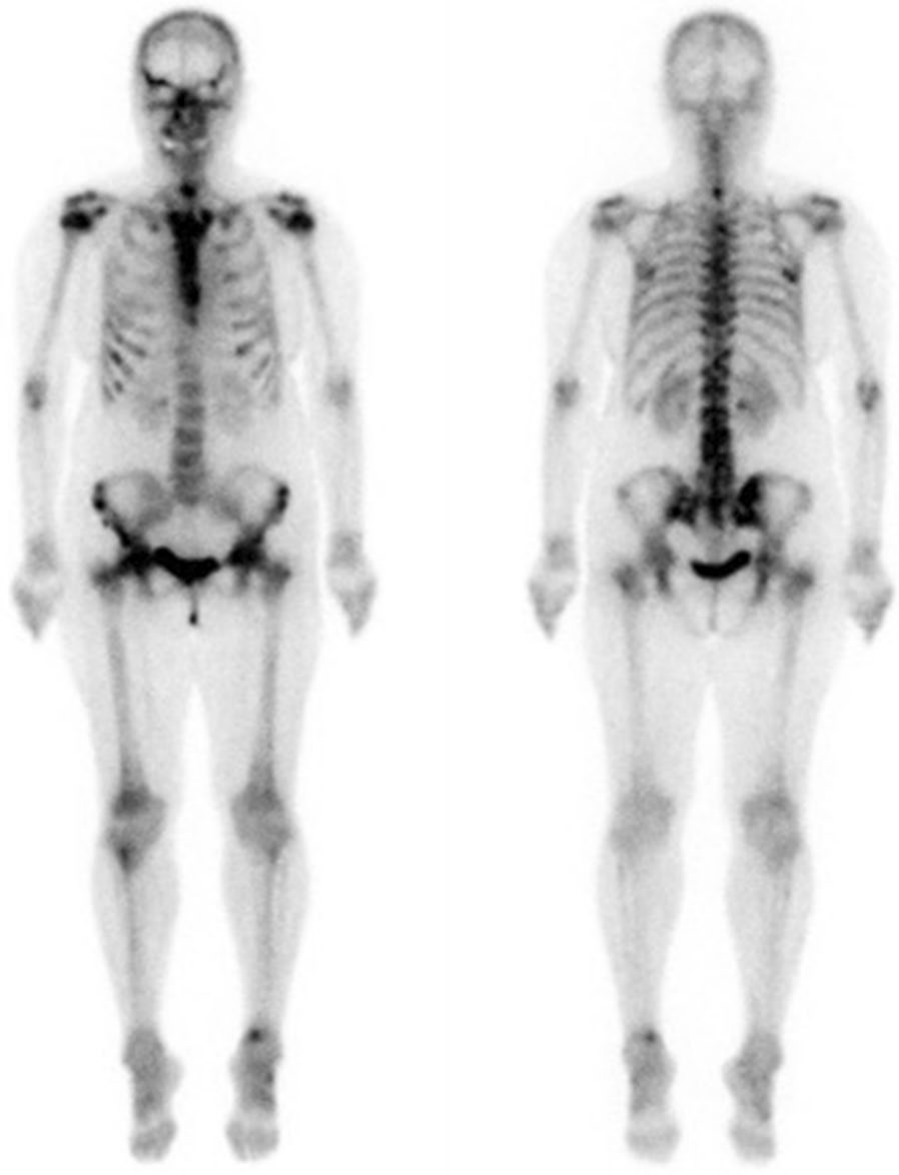
Bone scintigraphy was revealed uptake in the spine, ribs, and pelvis, however, there was no clear exacerbation compared to before MAHA onset

Systemic examination with CT showed no evidence of visceral metastasis, and bone scintigraphy revealed slight inhomogeneous uptake in the spine, ribs, and pelvis (Fig. 2); however, there was no clear exacerbation compared to previous results. Positron emission tomography-CT showed slight FDG uptake mainly in the spine and pelvis, but visceral metastasis was not identified. Although two bone-marrow biopsies were performed, dry tap occurred; thus, tumor cells could not be confirmed. The patient had difficulty walking because of severe anemia and required blood transfusions twice a week. The characteristic fragmented red cells, hemolytic anemia, and thrombocytopenia were observed; moreover, the underlying breast cancer, the synchronicity of the elevation of tumor markers, and the patient’s symptoms confirmed the diagnosis. Considering the circumstances mentioned above, we diagnosed the patient’s clinical features as CR-MAHA associated with multiple bone metastases, and chemotherapy with paclitaxel (80 mg/m^2^ weekly) was initiated.

**Fig. 3:**
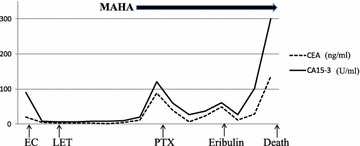
Transition of Tumor marker was linked with the treatment effect. *EC* epirubicin + cyclophosphamide, *LET* letrozole, *PTX* paclitaxel

**Fig. 4:**
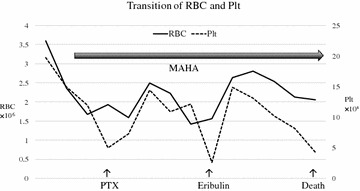
Transition of RBC and Plt counts was linked with the treatment effect. But in course of treatment, RBC transfusions were performed quite frequently. *RBC* red blood cell, *Plt* platelet

As the therapeutic effect was difficult to detect by imaging, this was determined based on the clinical symptoms and changes in tumor marker levels. Therapeutic course and changes in tumor marker levels, red blood and platelet counts are shown in Figs. 3 and 4.

Paclitaxel (PTX) was effective, with the tumor marker levels decreasing, the fragmented red cells in the peripheral blood disappearing, the frequency of blood transfusion reducing, and anemia improving. However, 3 months later, tumor marker levels increased again, fragmented red cells emerged in the peripheral blood, and anemia was exacerbated. Chemotherapy was switched to Eribulin and a temporary decrease in tumor marker levels and improvement in anemia were observed, but 3 months later, tumor marker levels increased again, with exacerbation of anemia and an increase in fragmented red cells.

Another switch in treatment was planned, but deterioration of liver function and exacerbation of hepatosplenomegaly were observed along with an elevation in tumor marker levels (CEA 29.0–135, CA15-3 102 to >300). CT and US did not show a space-occupying legion suggestive of liver metastasis. Liver failure developed and chemotherapy was discontinued. Best supportive care was selected and the patient died a month later. The survival period after MAHA onset was 13 months, and the overall survival after diagnosis was 41 months.

## Discussion and evaluation

Primary TMA syndromes are specific disorders that require specific treatments. They include TTP, HUS, and drug-induced TMA. Although these disorders have been studied separately in the past, in recent decades, they have been combined as common TMA syndrome, which includes definitive clinical and pathological features, MAHA, thrombocytopenia, and organ injury (James and Carla 2014). MAHA was first reported by Brain et al. (1962) as a form of hemolytic anemia marked by the emergence of fragmented red cells in peripheral blood (Brain et al. 1962). CR-MAHA is distinguished from primary TMA in terms of its underlying disorders and treatment options. CR-MAHA has been reported in association with several types of carcinoma since the 1980s (Pendse et al. 2014; Lechner and Obermeier 2012; Shin et al. 2011; Rauh et al. 2011; George 2011; Himmelmann and Schefer 2009; Ali et al. 2007; Arkenau et al. 2005; Yeh et al. 2002; Lockhart 2001; Fontana et al. 2001; Ataga and Graham 1999; Narita et al. 1997; Tsatsaris et al. 1996; Nordstrom and Strang 1993; Bastecky et al. 1992; Collins et al. 1991; Canellos and Mark 1984). Lechner,K et al. conducted a literature review of 168 reports of CR-MAHA; according to cancer type, gastric cancer was the most frequent, followed by breast, prostate, and lung cancers. Most cases were associated with a solid cancer, but malignant lymphoma was noted in some (Lechner and Obermeier 2012). MAHA often occurred in cases with advanced cancer associated with widespread metastasis or recurrence, but was also rarely reported in cases without metastasis. Of the 168 cases reviewed, 36 were of breast cancer (Lechner and Obermeier 2012).

We reviewed 51 cases of CR-MAHA associated with breast cancer (including the previously reported 36 and our present case) (Table 1) (Pendse et al. 2014; Lechner and Obermeier 2012; George 2011; Himmelmann and Schefer 2009; Lockhart 2001; Fontana et al. 2001; Ataga and Graham 1999; Narita et al. 1997; Nordstrom and Strang 1993; Bastecky et al. 1992; Collins et al. 1991; Canellos and Mark 1984; Brain et al. 1962). The histology of these cases was rarely reported, but invasive ductal carcinoma was the most common; there were two cases with invasive lobular carcinoma and one case with mucinous carcinoma. Thus, CR-MAHA onset does not seem to be linked to any specific histological type of breast cancer. Mucinous adenocarcinoma is considered the most common in other carcinomas. For these reason, in vitro studies have indicated a possibility of coagulation factor X activation by mucin, although the underlying mechanism remains unknown (Nordstrom and Strang 1993).

**Table 1:** 51 Reported cases of MAHA associated with breast cancer

Author	Age	Bone marrow metastasis	Intervention	Prognosis (survival time)	Notes
Pendse et al. (2014)	69	+	None	Soon death	
Kok et al. (2014)	43	Bone meta only	Discontinuation of GEM	Recovery	s/o GEM induced MAHA
Lechner and Obermeier (2012) (case reports review included 36 breast cancer cases)	Average 54 (19–82)	24/36	Each cases	4 months (chemotherapy) Half a month (no treatment)	
George (2011)	52	+	Plasma exchange	3 days	Death due to micro thrombi in multiple organs
Himmelmann and Schefer (2009)	66	–	None	10 days	
Lockhart (2001)	Unknown (middle age)	+	Chemotherapy	Survival for more than 1 year	
Fontana et al. (2001) (Case A)	69	+	Chemotherapy	No description of death	
Fontana et al. (2001) (Case B)	62	+	None	4 days	
Fontana et al. (2001) (Case C)	60	+	None	1 day	Death due to pulmonary embolism
Ataga and Graham (1999)	46	+	None	7 days	
Narita et al. (1997)	45	+	Chemoendcrinetherapy	No description of death	
Nordstrom and Strang (1993)	58	+	Chemotherapy	12 months	
Bastecky et al. (1992)	28	+	None	10 days	Death due to pulmonary embolism
Collins et al. (1991)	54	+	Chemotherapy and plasma exchange	No description of death	
N Engl J Med Case Record. (1984)	53	Unknown	Plasma exchange	4 days	Death due to tumor embolism in liver
Own case 2015	54	−	Chemotherapy	13 months	

The literature was extracted from 1984 to 2015

*GEM* gemcitabine

The reported age of onset ranges from 19 to 82 years, suggesting that onset can occur at any age (Pendse et al. 2014; Lechner and Obermeier 2012; George 2011; Himmelmann and Schefer 2009; Lockhart 2001; Fontana et al. 2001; Ataga and Graham 1999; Narita et al. 1997; Nordstrom and Strang 1993; Bastecky et al. 1992; Collins et al. 1991; Canellos and Mark 1984; Brain et al. 1962). CR-MAHA is rare, and detailed reports on its incidence according to cancer type are not available.

In our review, cancer cell infiltration into the bone marrow was observed in 36 of 51 cases (71 %). In the present case, although bone marrow biopsies resulted in dry taps, PET-CT findings suggested cancer cell infiltration into the bone marrow. This does not mean that MAHA develops more frequently in cases with bone marrow infiltration; the relationship between cancer cell infiltration into the bone marrow and MAHA remains unclear.

Very little is known about the pathophysiology of CR-MAHA; microvascular thrombi of carcinoma cells were found in multiple organs in many autopsy cases. The mechanism by which hemolysis progresses due to the mechanical fragmentation of red blood cells as blood flows through the vascular lumen narrowed by such tumor thrombi has been proposed (Lockhart 2001; Canellos and Mark 1984; Stephens 2011). Accordingly, rationale for the use of antineoplastic agents is supported, as an effort to reduce tumor burden is considered most important in improving MAHA.

MAHA is reportedly associated with TTP and HUS, with very similar pathological states for both; however, CR-MAHA is believed to have a different pathogenic mechanism (Lechner and Obermeier 2012; Lockhart 2001; Nordstrom and Strang 1993; Canellos and Mark 1984).

On the other hand, chemotherapy-induced MAHA has been reported as rare cases: Mitomycin C use has been implicated most frequently (George 2011; Bruntsch et al. 1984), and gemcitabine use has been reported recently (Kok et al. 2014). In cases of suspected chemotherapy involvement, some patients were reported to have recovered with symptomatic treatment such as discontinuation of the causative agent and blood transfusion (George 2011; Brain et al. 1962; Bruntsch et al. 1984).

Drug-induced MAHA and CR-MAHA are considered pathophysiologically distinct conditions. Chemotherapy is the only effective treatment for CR-MAHA, whereas plasma exchange is almost ineffective, with most patients dying within 2 weeks (1–10 days) (Pendse et al. 2014; Lechner and Obermeier 2012; George 2011; Himmelmann and Schefer 2009; Lockhart 2001; Fontana et al. 2001; Ataga and Graham 1999; Narita et al. 1997; Nordstrom and Strang 1993; Bastecky et al. 1992; Collins et al. 1991; Canellos and Mark 1984; Kok et al. 2014). In the study by Lechner et al. that included other carcinomas, the median survival of patients receiving chemotherapy was 4 months and that of patients receiving either symptomatic treatment or no treatment was 0.5 months (Lechner and Obermeier 2012). Response to chemotherapy was relatively favorable, and in some cases, survival for almost a year was achieved with the improvement of anemia (Lechner and Obermeier 2012; Lockhart 2001; Fontana et al. 2001; Nordstrom and Strang 1993). However, the prognosis in these cases was poorer than that of similar cases not complicated by MAHA (Lechner and Obermeier 2012).

The patient in the present case survived for 13 months after chemotherapy initiation. Therefore, following a CR-MAHA diagnosis, chemotherapy should be initiated as soon as possible, and the condition should be considered life threatening despite the lack of aggressive organ metastasis. In cases of breast cancer, hormone therapy is a treatment option, with some reports suggesting its efficacy (Narita et al. 1997), but the selection of chemotherapy instead seems desirable, because if hormone therapy is not successful, a switch to a different regimen might not be possible considering the rapid progression of the condition. Chemotherapy may have been effective, but the patient ultimately died of liver failure. The patient’s family refused an autopsy; thus, the cause of liver failure remains unclear. Microvascular tumor thrombi have been found in multiple organs in many autopsy cases involving MAHA, and resultant multiple organ failure was the cause of death in most cases. In the present case, the marked elevation in tumor marker levels and hepatomegaly and splenomegaly exacerbation were observed immediately before death, suggesting that liver failure was rapidly developing due to the tumor thrombi. This course is similar to that of the previously reported N Engl J Med case (1984).

Furthermore, in this present case, frequent blood transfusion was required to continue chemotherapy and maintain quality of life. The possibility of hemochromatosis consequent to excessive blood transfusion should be kept in mind, and the appropriate use of iron chelating agents should be considered.

Although MAHA is a clinical condition associated with a very poor prognosis, we consider it controllable for a long period by rapid introduction of chemotherapy in many cases. Early diagnosis based on the careful examination of the clinical condition and introduction of chemotherapy as early as possible are believed to contribute to prolonging life.

## Conclusions

MAHA is a nearly oncologic emergency that medical oncologists need to be able to recognize even though it rarely occurs in breast cancer.

## References

[CR1] Ali N, Kamran N, Adil S, Pervez S (2007). Metastatic signet ring gastric adenocarcinoma presenting with microangiopathic hemolytic anemia. Indian J Gastroenterol.

[CR2] Arkenau HT, Mussig O, Buhr T, Jend HH, Porschen R (2005). Microangiopathic hemolytic anemia (MAHA) as paraneoplastic syndrome in metastasized signet ring cell carcinomas: case reports and review of the literature. Z Gastroenterol.

[CR3] Ataga KI, Graham ML (1999). Microangiopathic hemolytic anemia associated with metastatic breast carcinoma. Am J Hematol.

[CR4] Bastecky J, Langr F, Chrobak L, Hlava A, Kvasnicka J (1992). Tumor microembolization in the lungs—a cause of marked dyspnea, syncope, hemolytic syndrome and disorders of hemocoagulation. Vnitr Lek.

[CR5] Brain MC, Dacie JV, Hourihane DO (1962). Microangiopathic haemolytic anaemia: the possible role of vascular lesions in pathogenesis. Br J Haematol.

[CR6] Bruntsch U, Groos G, Tigges FJ, Gallmeier WM (1984). Microangiopathic hemolytic anemia, a frequent complication of mitomycin therapy in cancer patients. Eur J Cancer Clin Oncol.

[CR7] Case Records of the Massachusetts General Hospital (1984). Weekly clinicopathological exercises. Case 26-1984: microangiopathic hemolytic anemia in a 53-year-old woman with metastatic breast cancer. N Engl J Med.

[CR8] Collins PW, Jones L, Pocock C, Newland AC (1991). Microangiopathic haemolysis associated with occult carcinoma. Clin Lab Haematol.

[CR9] Fontana S, Gerritsen HE, Kremer Hovinga J, Furlan M, Lammle B (2001). Microangiopathic haemolytic anaemia in metastasizing malignant tumours is not associated with a severe deficiency of the von Willebrand factor-cleaving protease. Br J Haematol.

[CR10] George JN (2011). Systemic malignancies as a cause of unexpected microangiopathic hemolytic anemia and thrombocytopenia. Oncology (Williston Park).

[CR11] Himmelmann A, Schefer H (2009). Microangiopathic haemolytic anaemia in a patient with metastatic breast cancer. Br J Haematol.

[CR12] James NG, Carla MN (2014). Syndrome of thrombotic microangiopathy. N Engl J Med.

[CR13] Kok VC, Wu SC, Lee CK (2014). Successful remission of hemolytic–uremic syndrome during the third-line weekly gemcitabine for metastatic breast cancer. Breast Cancer (Auckl).

[CR14] Lechner K, Obermeier HL (2012). Cancer-related microangiopathic hemolytic anemia: clinical and laboratory features in 168 reported cases. Medicine (Baltimore).

[CR15] Lockhart AC (2001). Microangiopathic haemolytic anaemia in metastatic malignancy. Hosp Med.

[CR16] Narita M, Nakao K, Ogino N, Emoto T, Nakahara M, Yumiba T (1997). A case of microangiopathic hemolytic anemia associated with breast cancer: improvement with chemoendocrine therapy. Breast Cancer.

[CR17] Nordstrom B, Strang P (1993). Microangiopathic hemolytic anemias (MAHA) in cancer. A case report and review. Anticancer Res.

[CR18] Pendse AA, Edgerly CH, Fedoriw Y (2014). Hemolytic anemia and metastatic carcinoma: case report and literature review. Lab Med.

[CR19] Rauh MJ, Al Habeeb A, Chang H (2011). Microangiopathic hemolytic anemia and leukoerythroblastic blood film heralding bone marrow metastatic gastroesophageal adenocarcinoma. Pathol Res Pract.

[CR20] Shin SY, Park H, Chae SW, Woo HY (2011). Microangiopathic hemolytic anemia as the first manifestation of metastatic signet ring cell carcinoma of unknown origin: a case report and review of literature. Korean J Lab Med.

[CR21] Stephens J (2011). Clinical tips: managing MAHA in oncology patients. Can Oncol Nurs J.

[CR22] Tsatsaris V, Lotz JP, Parrot A, Andre T, Lafont B, Bouleuc C (1996). Microangiopathic hemolytic anemia associated with uterine sarcoma: report of a case. Review of the literature. Rev Med Interne.

[CR23] Yeh KY, Dunn P, Chang JW, Liaw CC (2002). Microangiopathic hemolytic anemia in a patient with recurrent anal cancer and liver metastasis. Chang Gung Med J.

